# Sunlight-Induced Photocatalytic Removal of Paracetamol Using Au-TiO_2_ Nanoparticles

**DOI:** 10.3390/nano15050358

**Published:** 2025-02-26

**Authors:** Lamine Aoudjit, Joana M. Queirós, A. S. Castro, Djamila Zioui, Noelia González-Ballesteros, S. Lanceros-Mendez, Pedro M. Martins

**Affiliations:** 1 Unité de Développement des Equipements Solaires, UDES/Centre de Développement des Energies Renouvelables, CDER, Bou Ismail 42415, Algeria; aoudjit.lamine@udes.dz (L.A.); zioui.djamila@udes.dz (D.Z.); 2 Physics Centre of Minho and Porto Universities (CF-UM-UP) and LaPMET—Laboratory of Physics for Materials and Emergent Technologies, University of Minho, 4710-057 Braga, Portugal; id11653@alunos.uminho.pt; 3 Centre of Molecular and Environmental Biology, University of Minho, 4710-057 Braga, Portugal; pamartins@bio.uminho.pt; 4 IB-S—Institute for Research and Innovation on Bio-Sustainability, University of Minho, 4710-057 Braga, Portugal; 5 Centre of Chemistry, University of Minho, 4710-057 Braga, Portugal; 6 Departamento de Química Inorgánica, Universidade de Vigo, 36310 Vigo, Spain; noeliagb@uvigo.gal; 7 BCMaterials, Basque Center for Materials, Applications and Nanostructures, UPV/EHU Science Park, 48940 Leioa, Spain; 8 Ikerbasque, Basque Foundation for Science, 48009 Bilbao, Spain

**Keywords:** Au-TiO_2_ catalyst, paracetamol, photocatalytic degradation, solar light, water remediation

## Abstract

Using sunlight as the driving force for photocatalytic processes holds great promise for sustainability. As a starting point for developing a material capable of degrading aquatic pollutants using solar energy as a stimulus, this work focuses on synthesizing Au-TiO_2_ nanocomposites using the deposition–precipitation method. Characterization of Au-TiO_2_ nanoparticles was performed by X-ray diffraction (XRD), Fourier transform infrared spectroscopy (FTIR), and Transmission Electron Microscopy (TEM). A model pollutant, paracetamol, was used to test the synergetic effect of Au (0.05 wt%) nanoparticles (NPs) with TiO_2_ on photocatalytic activity. The influence of the parameters pH, loading (0.4, 0.8, and 1 g/L), pollutant concentration (20, 30, 40 ppm), and contact time (30, 60, 90, 120, 150, and 180 min) was studied by exposing the NPs to solar radiation. The photocatalytic degradation was most effective at a contact time of 3 h, an initial concentration of 20 ppm, and a pH of 6.8. Under these conditions, paracetamol in 1 g/L of Au-TiO_2_ nanocomposites can be degraded by more than 99.17% under solar irradiation. As a result of the Au-TiO_2_ composite’s ability to successfully serve as a photocatalyst using sun radiation, water purification processes can be more widespread, cost-effective, and environmentally friendly.

## 1. Introduction

Earth’s most valuable and critical natural resource is water, which is subject to high demand and contamination [1]. In recent times, the growth of industrial, agricultural, and residential activities has contributed to releasing numerous hazardous pollutants into wastewater, leading to continuous contamination of available freshwater sources [2]. The increasing sensitivity of analytical methods has revealed the presence of trace compounds such as pharmaceuticals [3], veterinary products [4], toiletries [5], food additives [6], pesticides [7], and other contaminants [8]. These compounds are typically known as Persistent Organic Pollutants (POPs). They are renowned for their toxicity and environmental persistence, which has raised significant concerns due to the potential threat to aquatic ecosystems and human health [9,10].

Pharmaceuticals are a significant source of pollution of water bodies, causing increasing concern due to their extensive use and unregulated discharge [11]. One compound of particular concern is acetaminophen (N-acetyl-p-aminophenol), widely known as paracetamol (PAR), which is a mild analgesic, readily available, and widely used to relieve headaches and other minor pains, among other treatments [11,12]. Municipal wastewater treatment plants (WWTPs) address PAR through traditional methods [13], making it one of the most prevalent compounds in natural and drinking water sources [12]. Conventional methods, such as biological treatment [14], ozonation [15], precipitation [16], reverse osmosis [17], and flotation/coagulation [18], have proven ineffective in removing pharmaceutical molecules owing to their high polarity and solubility in water, along with their molecular weight, structure, functionality, and variations in shape [19].

A recent analysis revealed the presence of PAR in surface water, groundwater, and tap or drinking water in 29 countries worldwide, with average and maximum measured environmental concentrations reaching 0.161 μg/L and 230 μg/L, respectively [20,21]. Considering the ubiquitous presence of PAR in water matrixes, several ecotoxicity studies have been conducted in different types of organisms. Daniel et al. [22], who assessed the acute and chronic effects of paracetamol at ecologically relevant levels on the crustacean *Daphnia magna*, reported no biochemical modifications after chronic exposure; however, high concentration levels of PAR are capable of triggering significant antioxidant responses. In another study, *Oncorhynchus mykiss* (rainbow trout) was shown to be highly reactive to PAR at ecologically relevant levels (12.5–50.0 μg/L), suffering oxidative effects and peroxidative damage [23]. Concerning plant ecotoxicity, a report shows alterations in plant physiology when exposed to PAR (2.5 g/L), such as in its uptake and absorption [24], metabolism and growth [25].

Considering the inefficiency of classical approaches to water remediation and the mentioned ecotoxicity impacts of PAR in the environment, it is paramount to foster the development of new materials and technologies that address this emerging problem. In this context, non-conventional treatments, including adsorption [26], membrane technologies [27], and Advanced Oxidative Processes (AOPs) [28], stand out for their potential to improve the removal of these contaminants from water [19]. Among all these approaches, AOPs, particularly photocatalysis, have attracted widespread interest due to the efficient use of solar energy to degrade organic pollutants [29]. This environmentally friendly process offers several benefits, including cost-effectiveness, use under ambient conditions, and efficient solar energy utilization to decompose organic pollutants [29,30,31,32,33,34].

The activation of semiconductors by solar light irradiation is a strategy to improve materials’ functional response [35]. The semiconductors used as photocatalysts predominantly include titanium dioxide (TiO_2_), which is the subject of in-depth research and widely applied in photocatalysis. This popularity is due to its outstanding optical and oxidation properties, superhydrophilicity, chemical stability, and low toxicity [36,37]. However, TiO_2_ exhibits certain limitations. These include higher hole/electron recombination rates and reduced efficiency under visible light [38]. To overcome these limitations, TiO_2_ is usually combined with other materials that attenuate electron–hole recombination, thus facilitating the transfer of these charges to electron acceptors and donors. Other strategies include introducing morphological modifications like increasing porosity or surface area or doping TiO_2_ with different elements [39]. Noble metals such as silver (Ag) [40], platinum (Pt) [41], palladium (Pd) [42], and gold (Au) [43] can improve photocatalytic efficiency under visible light by acting as an electron trap, promoting interfacial charge transfer and therefore slowing down electron–hole pair recombination. Gold nanoparticle functionalization has attracted considerable attention because of its stability and nontoxicity [44,45,46,47]. In Au/TiO_2_ composites, Au absorbs visible light through localized surface plasmon resonance (LSPR). This phenomenon allows Au nanoparticles to absorb visible light, thereby generating electron–hole pairs that can be injected into the conduction band of TiO₂, thus enhancing its photocatalytic performance [48,49]. Additionally, they have proven effective as photocatalysts in contaminant degradation processes using visible light [50]. This system is, therefore, promising for applications in photocatalytic reactions in water remediation [40,41,51,52].

This research focused on the synthesis and physical–chemical characterization of the Au-TiO_2_ and a systematic assessment of their efficiency in the degradation of PAR, aiming to identify the optimal experimental parameters (i.e., PAR initial concentrations, catalyst concentration, pH, and type of radiation) for achieving the most effective photocatalytic activity. Moreover, the functionalization of the material was designed to improve its ability to absorb visible light, thereby enhancing its efficiency under solar radiation. Conducted in Algeria, this study capitalizes on the country’s geographical position as one of the regions with the highest solar energy potentials globally, benefiting from abundant solar exposure [53]. By utilizing this natural resource, the approach also emphasizes the reuse of the material over multiple cycles and explores the process’s scalability, striving to combine cost-effectiveness with sustainability.

## 2. Experiment

### 2.1. Materials and Methods

P25 TiO_2_ nanoparticles were provided by Evonik (Essen, Germany), and Gold(III) chloride trihydrate was purchased from Sigma-Aldrich (St. Louis, MI, USA). Sodium hydroxide (98% purity) was purchased from Biochem Chemopharma Company (France). Paracetamol (C_8_H_9_NO_2_, ≥99%) was purchased from Merck (Darmstadt, Germany), and some of its most relevant characteristics and properties are shown in Table 1. All solutions were prepared with ultrapure water (Millipore Milli-Q—resistivity 18.2 MΩ·cm).

### 2.2. Synthesis of Au-TiO_2_ Nanocomposite Photocatalysts

The Au-TiO_2_ nanocomposites were synthesized by a deposition–precipitation method described by the authors [47,51,52,54,55]. Briefly, 200 mg of TiO_2_ nanoparticles was dispersed in 40 mL of ultrapure water (UP) in a sonication bath for 30 min. Afterward, adding 1600 μL of chloroauric acid solution (HAuCl_4_), an Au loading of 0.05 wt% was obtained. The solution was stirred at 60 °C for 10 min to ensure homogeneous dispersion of the gold precursor. The pH was adjusted to approximately 9 by adding a sodium hydroxide solution (NaOH) from Sigma Aldrich (St. Louis, MI, USA) and mixing for 10 min. The solution was centrifuged for 20 min before being twice washed with ultrapure water (UP) and subjected to a 5 min ultrasonic process. Finally, the nanocomposite was dried overnight at 80 °C (J.P Selecta from Barcelona- Spain) in an oven and then ground with a mortar and pestle to obtain a fine powder.

### 2.3. Au-TiO_2_ Nanocomposite Characterization

The morphological characterization of the Au-TiO_2_ nanocomposites was carried out by transmission electron microscopy (TEM) using a JEOL JEM 1010 microscope (JEOL, Tokyo, Japan) operated at an accelerating voltage of 100 kV. Samples were first dispersed in water and sonicated to ensure uniform dispersion. Subsequently, the material was deposited onto a 400-mesh copper grid coated with Formvar and carbon using the drop-casting method. The crystallographic phases of the Au-TiO_2_ nanocomposites were evaluated by X-ray diffraction (XRD) using a Bruker D8 Discover diffractometer with incident CuKα radiation (40 kV and 30 mA) from Bruker (Billerica, MA, USA). Fourier-transformed infrared spectroscopy (FTIR) was carried out in the range of 500–4000 cm^−1^, 64 scans with a resolution of 4 cm^−1^, using an FTIR Alpha instrument (Bruker Corporation, Billerica, MA, USA) to determine the chemical stability of the nanocomposites. The UV-vis reflectance measurements were performed using a Shimadzu UV 2501-PC (Kyoto, Japan) with and integrating sphere to evaluate the optical properties of both pristine TiO_2_ and the Au-TiO_2_ nanocomposite.

### 2.4. Photocatalytic Degradation Processes

**Figure 1:**
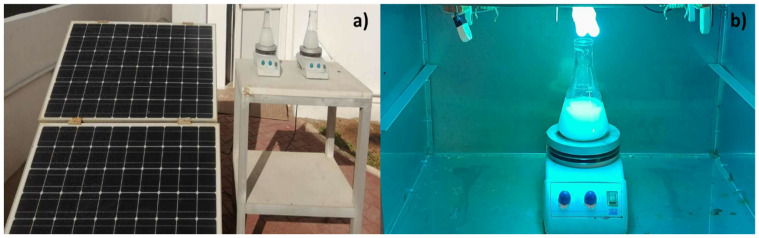
Photographs of the experimental setup to assess the degradation of PAR using Au-TiO_2_ nanoparticles: (**a**) under solar radiation, where the Erlenmeyer contains the contaminant solution and nanoparticles while being magnetically stirred and powered by a solar panel that collects clean sunlight energy; (**b**) under a UV lamp.

The photocatalytic degradation of PAR was conducted in a batch photoreactor, represented by a 250 mL open Erlenmeyer. Before the photocatalytic tests, the Au-TiO_2_ nanocomposite was kept in the dark for 30 min to ensure the adsorption–desorption equilibrium. The solution was then irradiated with sunlight (northern Algeria—latitude 36°39′; longitude 2°42′ at sea level—map location in Figure S1 in Supplementary Material) and UV radiation under magnetic agitation for three hours, and 3 mL aliquots were withdrawn at 30 min intervals, as illustrated in Figure 1a.

Concurrently, the procedure mentioned above was conducted, subjecting the solution to ultraviolet radiation (UV), which was quantified with a pyranometer (KIPP & ZONZN, CMP11, Sterling, VA, USA) with a spectral range from 285 to 2800 nm (800 W/m^2^). The effect of UV radiation was investigated using a Philips PL-L 24W/10/4P UV lamp with a wavelength of 365 nm (spectra in Supplementary Materials—Figure S3) and an irradiance of 18.6 W/m^2^ (Figure 1b). A graph showing the variation of solar intensity over the course of the day is provided in the Supplementary Materials (Figure S2), offering additional context to the experimental conditions.

This photocatalytic study examined several parameters, including the (1) effect of photolysis, adsorption, and photocatalysis on the removal of paracetamol from the solution, the (2) influence of the initial concentration of paracetamol, the (3) effect of the Au-TiO₂ dosage, the (4) pH of the paracetamol solution and the (5) type of radiation. All the parameters used are described in Table 2.

All withdrawn samples were analyzed using a UV–visible spectrophotometer (Shimadzu-1800, Kyoto, Japan), and the peak at 243 nm was used to monitor paracetamol absorbance over the irradiation time. The pollutant concentration was determined using a calibration curve (Figure S5 in the Supplementary Materials), and the degradation percentage was estimated using Equation (1) [56]:

(1) $$Degradation\;\left(\%\right)=\frac{C_{0}-C_{t}}{C_{0}}\times 100$$

where *C_o_* is the initial pollutant concentration, and *C_t_* is the concentration after a given reaction time *t* (min).

Still related to the photocatalytic degradation, the nanoparticles’ reusability was evaluated based on their effectiveness over three usage cycles. The same catalyst previously employed was resuspended in distilled water after each cycle (180 min), recovered through centrifugation, and dried at room temperature.

### 2.5. Chemical Oxygen Demand Measurements

Samples were withdrawn at different times during the PAR degradation process to ensure that the mineralization process was monitored. Chemical Oxygen Demand (COD) analysis was performed by boiling an excess of potassium dichromate (K_2_Cr_2_O_7_) in an acidic medium in the presence of silver sulphates (Ag_2_SO_4_) and mercury sulphate (HgSO_4_) as catalysts. The organic matter partially reduces the dichromate, and the remaining rate, expressed in percentage (%), can be determined by the following equation [29,33,57]:

(2) $$COD\left(\%\right)=\frac{{COD}_{0}-{COD}_{t}}{{COD}_{0}}\times 100$$

where COD_0_ and COD_t_ are the initial COD and COD at time t, respectively. The COD measurements were performed using a spectrophotometer DR 1900 LANGE HACH (Dusseldorf, Germany).

## 3. Results and Discussion

### 3.1. Characterization of Au-TiO_2_ Nanoparticles

**Figure 2:**
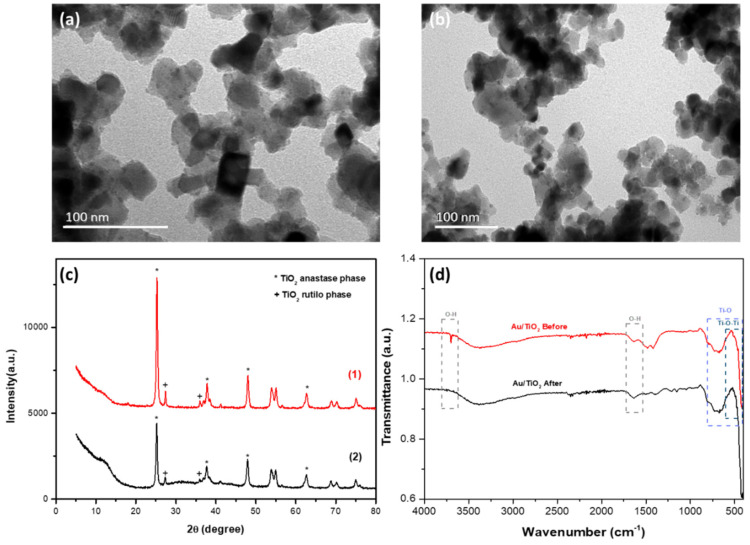
Scanning electron microscopy images of Au-TiO_2_ nanoparticles (**a**) before contact and (**b**) after contact with the paracetamol solution; (**c**) XRD pattern of Au-TiO_2_ before (1) and after (2) paracetamol degradation; (**d**) FTIR spectra of Au-TiO_2_ before and after paracetamol degradation.

The TEM technique was used to visualize the size, morphology and distribution of Au nanoparticles on the surface of TiO_2_ nanoparticles. The titanium catalysts were functionalized with gold particles obtained at 60 °C with an Au load of 0.05 wt%. Figure 2a shows a homogeneous distribution of Au nanoparticles (round-shaped darker spots) over TiO_2_, with a size of 3 nm (measured with Image J software, version 1.54g—200 nanoparticles), in agreement with the study by Martins et al. [47]. In addition, by comparing Figure 2a,b, it is shown that the contact with the paracetamol solution did not cause significant alterations in the morphology of the nanoparticles.

X-ray diffraction was carried out to assess the crystal structure of the Au-TiO_2_ nanocomposite. The samples in Figure 2c show the typical reflections of anatase (peaks at 25.3, 37.8, 48.0 and 62.6 degrees) and rutile (peaks at 27.49 and 35.99 degrees), in good agreement with the literature [45,51,58,59]. No significant differences existed between the intensities or positions of the diffraction peaks of the two samples before (1) and after (2) contact with the paracetamol solution during the degradation process. In addition, the nanocomposite did not undergo significant changes in its crystalline structure after the radiation and contaminated water exposure, confirming its high stability.

The FTIR spectrum of the Au-TiO_2_ nanocomposite shows peaks characteristic of the TiO_2_ compound. These include the Ti-O stretching vibrations in the 400–800 cm^−1^ range [60] and the Ti-O-Ti bending modes in the 400–600 cm^−1^ region [61]. In Figure 2d, distinct bands are observed in spectra at 3699 and 1638 cm^−1^, attributed to the O-H groups’ stretching vibrations and the water molecules’ deformation mode [62]. After the degradation of paracetamol, a certain amount of surface hydroxyl groups was removed by condensation and elimination of water due to exposure to sunlight during the degradation process of the Au-TiO_2_ nanocomposite.

To compare the differences in photocatalyst performance between TiO_2_ and Au-TiO_2_, their optical properties were investigated using UV-vis diffuse reflectance spectroscopy, as illustrated in Figure S4 of the Supplementary Material. Pristine TiO_2_ demonstrated high reflectance (95%) in the visible range (400–700), while the Au-TiO_2_ nanocomposite exhibited lower reflectance (64%), with a minimum (44%) at 545 nm, indicating the surface plasmon resonance, typically observed between 520 nm and 560 nm [63,64]. The broader absorption spectrum of the nanocomposite is thus attributed to its characteristic purple/pink coloration [65].

### 3.2. Photocatalytic Degradation of Paracetamol

**Figure 3:**
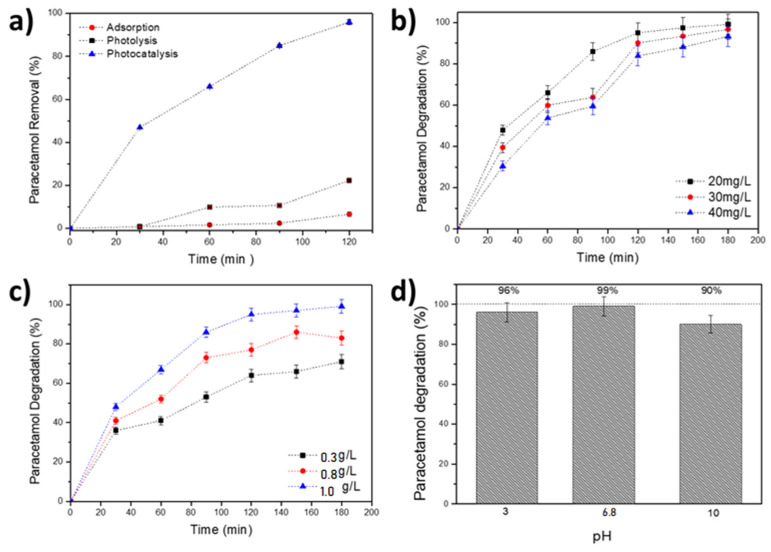
Study of photodegradation of paracetamol using sunlight radiation: (**a**) control process with C_PAR_ = 20 mg/L, C_catalyst_ = 1 g/L and free pH 6.8, for 120 min; (**b**) effects of initial concentration (20, 30 and 40 mg/L); (**c**) different doses of Au-TiO_2_ catalyst (0.3, 0.8 and 1.0 g/L); (**d**) impact of pH in solution (pH = 3, pH = 6.8 and pH = 10).

The photocatalytic performance of the prepared Au-TiO_2_ particles was investigated by monitoring the concentration of paracetamol (C_initial_ = 20 mg/L, pH 6.8, C_catalyst_ = 1 g/L) over 120 min when exposed to sunlight. Control experiments were conducted using TiO_2_ nanoparticles to evaluate paracetamol degradation under both solar and UV radiation, and the results are presented in Figure S3 of the Supplementary Materials. Additionally, preliminary studies were conducted to determine paracetamol stability (1), particle interaction in the absence of radiation (2), and Au-TiO_2_ photocatalytic performance (3). In the first scenario, a paracetamol solution was subjected to solar radiation (photolysis). In the second, particles were in contact with paracetamol without radiation (adsorption phenomenon). Lastly, in the third scenario, the particles were exposed to energetic radiation while coming into contact with paracetamol (photocatalysis). The outcomes are illustrated in Figure 3a.

**Figure 4:**
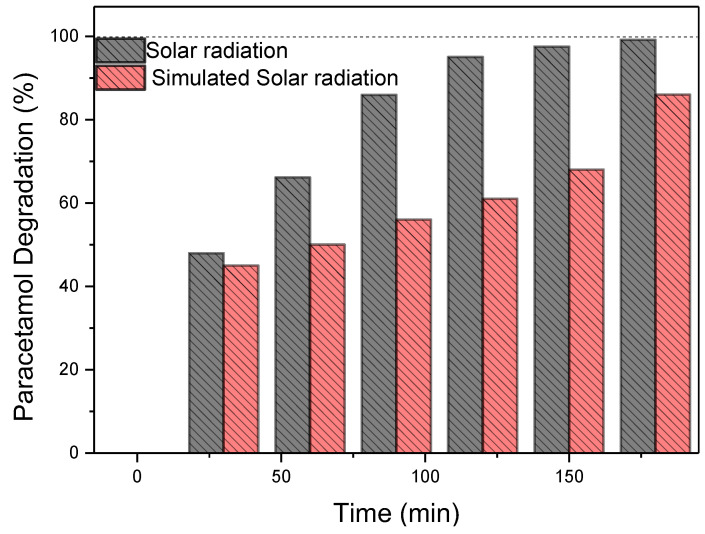
The impact of the irradiation source on the photocatalyst’s degradation of PAR in 180 min (C_PAR_ at 20 mg/L, C_catalyst_ at 1 g/L, and a pH of 6.8).

The impact of various parameters on the catalytic process was also evaluated, including initial concentration (20, 30, and 40 mg/L) (Figure 3b), catalyst dosage (0.3, 0.8, and 1.0 g/L) (Figure 3c), pH levels (3, 6.8 and 10) (Figure 3d) and the effect of radiation source: solar radiation located in northern Algeria (latitude 36°39′; longitude 2°42′ at sea level), from June to August, and UV radiation (Figure 4). These experiments were conducted to discern the influence of these factors on the degradation process of paracetamol.

#### 3.2.1. Influence of Parameters on the Photocatalytic Degradation of Paracetamol

The photocatalytic activity of the Au-TiO_2_ nanocomposite was evaluated by adsorption, photolysis, and photocatalytic degradation of paracetamol as a function of time. Figure 3a shows that the photocatalysis process allows up to 99% of paracetamol to be degraded after 120 min. At the end of 50 min, around 50% of the contaminant had been degraded.

On the other hand, the degradation rates of the adsorption and photolysis processes were not as efficient as photocatalysis, but adsorption showed a significant value of 22.31%. Photolysis showed a 6.61% rate of paracetamol degradation after 120 min. These results align with other studies [33,36,66] that identify heterogeneous photocatalysis as a method that can effectively oxidize most organic pollutants. Therefore, this study will be based on removing paracetamol by photocatalysis.

The initial pollutant concentration (C_0_) effect on the photocatalytic degradation efficiency of paracetamol was examined by varying the concentration within the range of 20 to 40 mg/L at pH (6.8) and a dose of 1 g/L Au-TiO_2_ nanoparticles. The variation in the paracetamol concentration was considered regarding the record in surface waters passing through pharmaceutical factories of 50 mg/L [66]. The degradation of paracetamol increases significantly at lower initial concentrations, as shown by the percentage curves in Figure 3b over 180 min. At an initial concentration of 20 mg/L, a minimum concentration of approximately 0.26 mg/L (99.17% degradation yield) was reached, while at 30 and 40 mg/L, the final concentrations were 0.99 mg/L and 2.64 mg/L, corresponding to degradation yields of 98.9% and 93.33%, respectively.

These results indicate a marginal discrepancy in the final PAR concentration across the varying initial concentration levels, suggesting that degradation efficiency slightly decreases as the pollutant concentration increases. Our results corroborate prior research [56,67] focused on removing emerging contaminants. The postulate has been made that high concentrations of pollutants obstruct the formation of free radicals (OH^−^) on the photocatalyst’s surface by coating active sites with pollutant molecules. This phenomenon hinders the formation of active species responsible for the photocatalytic reactions. In addition, high concentrations prevent light from penetrating, as opposed to low concentrations, which act as a barrier. Hence, the initial pollutant concentration correlates inversely with the degradation efficiency [29,31,36,68].

Various tests with different amounts of Au-TiO_2_ catalyst (0.4, 0.8, and 1 g/L) were carried out for photocatalytic studies to better understand the effect of catalyst dosage on paracetamol photocatalysis. Figure 3c illustrates the degradation rate of PAR following a 180 min reaction in the presence of different amounts of catalyst.

The results show that the degradation rate increases as the concentration of the Au-TiO_2_ catalyst increases. At 1 g/L Au-TiO_2_ dosage, the yield exceeds 99.17%. Thus, the photodegradation yield proportionally increases with the amount of the catalyst. The increased degradation rate is ascribed to increased active sites, which generate free radicals [30,68].

The effect of pH on the kinetics of paracetamol degradation was studied by varying the pH of the solution (3, 6.8, 10) (Figure 3d). The pH of the solutions was maintained at a constant level by adding sodium hydroxide (NaOH) as a basic medium and hydrochloric acid (HCl) as an acidic medium. The experimental outcomes indicate that pH moderately impacts paracetamol degradation, with a neutral pH (6.8) showing the highest degradation rate of 99.17% within 180 min. Moreover, the study demonstrated that PAR continued to degrade regardless of the pH of the solution, exhibiting a degradation efficiency of 90.00% at an alkaline pH of 10 and 96.69% at an acidic pH of 3 within the same time frame. The hydroxyl radical (OH·) is a powerful oxidizing agent of organic pollutants during the photolysis process, with its generation being dependent on pH. The production of OH· is slightly favored by alkaline conditions due to the increased availability of hydroxide ions. However, this effect is counterbalanced by an increase in electrostatic repulsion between the TiO_2_ surface and paracetamol molecules as pH rises (pKa = 9.38), which results in a marginal reduction in adsorption and, consequently, in degradation efficiency. In acidic conditions (pH < 6.3), the TiO_2_ surface carries a positive charge, which may enhance the adsorption of paracetamol.

As pH increases, however, the negative charge on the TiO_2_ surface strengthens, slightly hindering the adsorption of paracetamol molecules. This, combined with a reduction in certain reactive species, such as O_2_^−^·, due to the lower rate of oxygen reduction at high pH, may contribute to the observed decline in degradation efficiency. It is important to note that paracetamol degradation still occurs regardless of the pH level of the solution. A pH level of neutrality was selected as the optimal pH for the study at hand [69,70].

#### 3.2.2. Radiation Type

The effect of varying the source UV radiation and natural solar radiation on the degradation of the contaminant with Au-TiO_2_ nanoparticles was evaluated. The test was conducted using optimized parameters based on results obtained previously. Thus, the initial concentration of PAR was 20 mg/L, with 1 g/L of Au-TiO_2_ NP at a pH of 6.8. Figure 4 shows the results obtained over 180 min under solar irradiation (800 W/m^2^), where a degradation efficiency of approximately 99% was observed. Under UV radiation, significant degradation was also achieved, with efficiency reaching 86%. The degradation process is influenced by multiple factors, including radiation intensity and wavelength distribution, which affect the activation of electron–hole pairs and overall photocatalytic activity [36,56,68].

Solar radiation, which encompasses both highly energetic short ultraviolet wavelengths (<300 nm) and visible light, provides sufficient energy to activate TiO_2_ and facilitate the generation of hydroxyl radicals, thereby significantly enhancing degradation efficiency [71].

Control experiments using TiO₂ were performed under both solar and UV radiation to assess the impact of Au functionalization (Figure S6). Under solar irradiation, TiO₂ exhibited ~47% degradation within 180 min, whereas Au-TiO₂ achieved 99% degradation. A similar trend was observed under UV light, where TiO₂ led to ~40% degradation, while Au-TiO₂ exhibited a higher efficiency of 82%. The enhanced performance of Au-TiO₂ under solar irradiation can be attributed to the localized surface plasmon resonance (LSPR) effect of gold, which extends light absorption into the visible range and improves charge separation, ultimately boosting photocatalytic efficiency [72,73]. Additionally, gold nanoparticles contribute to reducing electron–hole recombination, further enhancing the photocatalytic efficiency of the system [52].

Based in the literature, the degradation mechanism involves the generation of hydroxyl radicals as primary oxidizing agents, regardless of the radiation source [74,75]. The enhanced efficiency under solar radiation may be attributed to a higher production of these radicals, driven by its broader spectrum and higher intensity. Initially, hydroxylation of the aromatic ring leads to the formation of intermediates such as 4-acetamidocatechol (4-AC) and 4-acetamidoresorcinol (4-AR), along with hydroquinone (HQ). These intermediates undergo successive oxidation and ring cleavage, yielding smaller carboxylic acids before complete mineralization. Under UV radiation, competitive pathways may arise, leading to alternative degradation intermediates and influencing the overall efficiency [76]. This mechanistic insight explains the superior performance of Au-TiO₂ under solar radiation compared to UV exposure.

The results demonstrate the potential of Au-TiO₂ as an efficient photocatalyst, particularly in real-world applications where natural sunlight is the primary energy source. The localized surface plasmon resonance (LSPR) effect of gold allows for better solar spectrum utilization, enhancing efficiency and making the system viable for large-scale environmental applications. The study carried out in Algeria—one of the sunniest countries in the world with an abundance of solar energy resources [53]—took advantage of the plentiful solar energy available, making the process highly profitable. This feature positions the photocatalytic system as an optimal, sustainable solution for addressing local contamination challenges [77,78]. By leveraging sunlight, a cost-free, renewable resource, the approach further enhances economic viability, aligning efficiency with environmental and cost-conscious practices. Furthermore, the intensity of solar radiation during the experimental day, as illustrated in Figure S2, was measured to provide a more accurate reflection of real-world conditions. Solar radiation is dynamic, varying over the course of the day, and this directly influences the photocatalytic performance. The literature contains numerous studies that have explored the use of sunlight as a primary energy source for the photocatalytic degradation of PAR, as seen in Table 3.

Table 3 presents a selection of studies focusing on the functionalization of semiconductors to enhance their ability to absorb visible radiation to degrade PAR in an aqueous solution. Direct comparisons in photocatalytic experiments are challenging due to the inherent complexity of these systems, which are conducted under varying conditions, including pH, contaminant concentration, nanoparticle dosage, and radiation intensity/wavelength. While this complexity hinders direct result comparisons, the data remain valuable for contextualization. Thus, in one of the studies, Bouarroudj and colleagues [67] achieved a paracetamol degradation rate of 97.00%, marginally lower than the rate obtained in this investigation. In this study, Ce/Ag-co-doped ZnO composites were used and employed for 180 min to degrade an initial pollutant concentration of 20 mg/L.

For instance, the work developed by Ivanova et al. indicates a marked enhancement in the degradation efficiency of ZnO when compared with its functionalization with Ag. This increase was more than twofold, from 40% to 83%. This improvement was evident when both materials were subjected to solar radiation and UV lamps [73]. The works from Table 3 show different approaches to functionalizing semiconductors to absorb a range of radiation, resulting in a degradation time of less than four hours, yielding higher than 55% degradation percentages.

Another relevant study explores the photocatalytic activity of Zr-WO_3_ for degradation of 20 mg/L of PAR under UV irradiation (254 nm, 25 W), achieving a degradation efficiency of 73% within 210 min. In comparison to the 86% degradation achieved in our Au-TiO_2_ system under UV light in a shorter reaction time, our photocatalyst demonstrated a higher efficiency under similar conditions [81]. Regarding the mentioned works, our material also presents high efficiencies (for similar conditions). However, our work systematically evaluates different parameters, such as the type of radiation and reusability, making it more consistent and robust. Additionally, the materials we employ, namely TiO_2_ and Au, represent a sustainable approach regarding aquatic ecosystems’ ecotoxicity compared to materials such as ZnO and Ag.

### 3.3. Chemical Oxygen Demand Analysis

**Figure 5:**
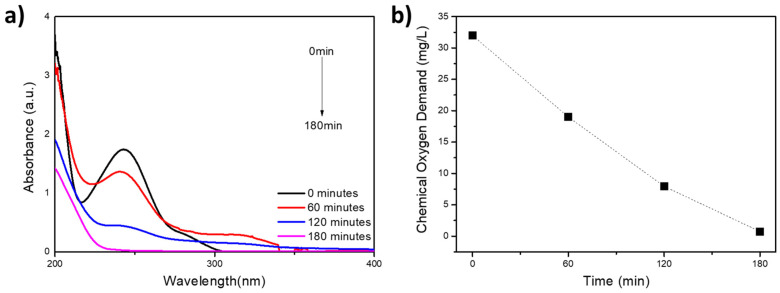
(**a**) UV–Vis spectra of paracetamol degradation under different sun irradiation times at pH = 6.8; (**b**) chemical oxygen demand (COD) at different times.

Chemical Oxygen Demand (COD) measures the oxygen required to oxidize organic compounds in water chemically. It assesses the quantity of oxygen required for the oxidation of organic matter present in wastewater and the overall oxygen consumption by said organic matter. This feature is of particular relevance and can be readily quantified, thereby facilitating the characterization of a range of waterbodies, including sewage, industrial waste, and treatment plant effluent [82]. At equivalent paracetamol degradation times (as illustrated in Figure 5a), the oxygen demand was calculated over 180 min (Figure 5b).

Figure 5a shows the absorption intensity of paracetamol throughout the photocatalyst degradation assay. It is possible to observe that the peak at 243 nm diminishes over 180 min of solar radiation exposure, indicative of the fragmentation of the primary PAR molecule. Complementing this, Figure 5b presents COD results, with values declining from 32 mg/L to 0.72 mg/L, confirming the mineralization of degradation by-products. The 99.17% degradation rate corresponds to the reduction of paracetamol. In comparison, the 98.53% mineralization rate reflects the near-complete conversion of intermediates into simpler compounds, demonstrating the effectiveness of the process in eliminating PAR. These results are consistent with those of Bouarroudj et al. [67], wherein the degradation percentage reached 97% within the corresponding time frame.

### 3.4. Reusability of the Au-TiO_2_ Nanocomposite

**Figure 6:**
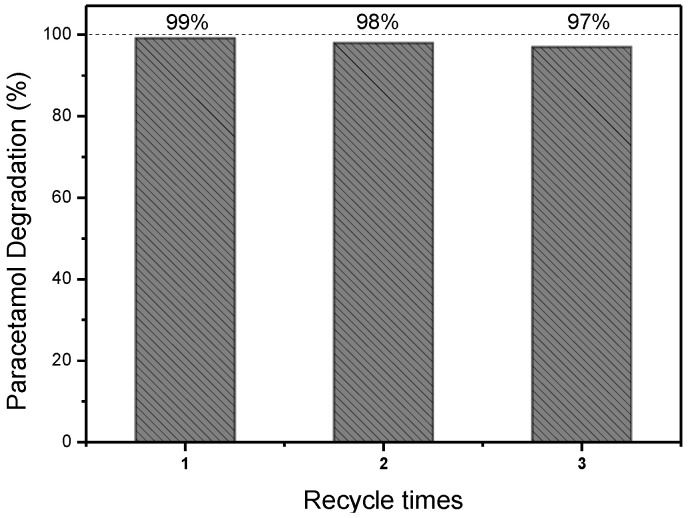
Photodegradation of paracetamol over three catalytic cycles in sunlight (C_PAR_ = 20 mg/L, C_catalyst_ = 1 g/L, and pH 6.8).

Reusability constitutes an essential material feature in sustainability and the circular economy. To evaluate the reusability of Au-TiO_2_ nanoparticles in the photocatalyst degradation of PAR under solar radiation, the nanoparticles were recovered and reused in two new cycles, as shown in Figure 6.

The results indicate that Au-TiO_2_ continues to exhibit a remarkable photocatalytic performance after three consecutive cycles with an efficiency loss below 2% [43]. This slight decrease in photocatalytic degradation efficiency can be attributed mainly to the residual loss of photocatalysts during the nanoparticle recovery through centrifugation and rinsing steps [83]. These outcomes are similar to reports in studies involving different catalysts. For context, ZnO nanoparticles demonstrated no substantial decline in photocatalytic activity after five cycles [84], and ZnO co-doped with Ce/Ag exhibited a decline from 97% to 95% after three reuse cycles [67], while another study reported a reduction from 98.4% to 94% [85]. In contrast, the Au-TiO_2_ photocatalyst demonstrated superior stability, maintaining higher efficiency over several cycles with minimal loss. This reinforces its stability, making it particularly advantageous for environmental remediation applications. Moreover, it indicates that the process can be scaled up, as the catalyst retains high activity even after recovery and reuse, ensuring a cost-effective and environmentally friendly approach for large-scale applications.

The system has demonstrated significant efficiency in PAR removal under the studied conditions, achieving complete degradation, with the process being driven primarily by the photocatalyst under solar radiation. However, to further enhance reusability, reduce operational costs, and minimize potential environmental impacts associated with nanoparticle dispersion, future work will focus on immobilizing these photocatalysts into polymeric substrates. This strategy has been successfully employed in previous studies, improving catalyst recovery and stability while maintaining high photocatalyst efficiency [54,86].

## 4. Conclusions

The efficient removal of PAR from aquatic environments is a critical challenge, requiring innovative, economically viable techniques and environmentally sustainable solutions. This study successfully developed Au-TiO_2_ nanocomposites using the deposition–precipitation technique and demonstrated their exceptional performance in the solar-powered degradation of PAR. A significant strength of this work lies in the comprehensive characterization of the nanocomposite using TEM, XRD, and FTIR, which confirmed their structural stability and chemical integrity, even after exposure to the contaminant and degradation process.

The photocatalytic activity of Au-TiO_2_ was optimized by meticulously evaluating key parameters, including the PAR concentration, catalyst dosage, pH value, and application of different radiation sources. Under optimal conditions (20 mg/L PAR, 1 g/L AuTiO_2,_ pH 6.8, and 180 min of sunlight exposure), the nanocomposite achieves an outstanding degradation efficiency, reducing the PAR initial concentration to 0.25 mg/L (99% efficiency). Photocatalysis played the dominant role compared to adsorption, which removed 4.95 mg/L (22.31%), and photolysis, which contributed to a reduction of 1.46 mg/L (6.61%). Notably, the functionalization of TiO_2_ with Au nanoparticles significantly enhanced the photocatalytic performance. Under solar radiation, the nanocomposite achieved 99% degradation, while 86% degradation was observed under UV exposure. This improvement is attributed to the LSPR of Au, which extends light absorption into the visible spectrum. Furthermore, the nanocomposite exhibits excellent stability during reuse, maintaining over 98% of its photocatalytic efficiency after three consecutive cycles, emphasizing its recyclability and cost-effectiveness for long-term applications.

The findings of this study highlight the potential of Au-TiO_2_ to act as a resilient, effective, and recoverable photocatalyst, thereby establishing it as a promising contender for addressing pharmaceutical contamination in water bodies. The study also establishes a robust framework for advancing sustainable and scalable water purification technologies, paving the way for effective and environmentally friendly solutions.

## Figures and Tables

**Table 1:** Characteristics and properties of paracetamol (PubChem).

Name IUPAC	Chemical Structure	λ _max_ Absorption (nm)	Molar Mass g/mol	UV–Vis Absorption Spectrum	Water Solubility (mg/mL) at 25 °C	Density (g/cm^3^)	p*k_a_*
N-(4-hydroxyphenyl)acetamide	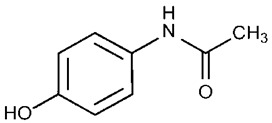	243	151.16	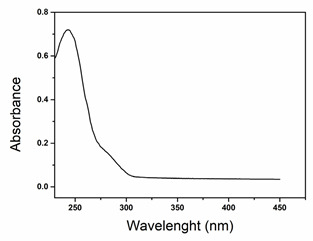	14.00	1.29	9.0–9.5

**Table 2:** Experimental parameters for the photocatalytic degradation of paracetamol, including variations in initial concentration, Au-TiO_2_ dosage, pH solution, and types of radiation exposure (natural sunlight and solar ultraviolet radiation).

	(1) Photolysis/Adsorption/Photocatalysis	(2) Paracetamol Initial Concentration	(3) Au-TiO_2_ Dosage	(4) pH Solution	(5) Type of Radiation
Time (min)	120	180	180	180	180
C_initial_[PAR] (mg/L)	20	20, 30, 40	20	20	20
C_dosage_[Au-TiO_2_] (g/L)	1	1	0.3, 0.8, 1	1	1
pH	6.8	6.8	6.6	3, 6.8, 10	6.8

**Table 3:** Comparison of studies on the efficiency of paracetamol degradation using different photocatalysts.

Material	C_PAR_ (mg/L)	Radiation	Time (Minutes)	Degradation (%)	References
Ce/Ag co-doped ZnO	20	Solar light	180	97.00	[51]
TiO_2_-Au	20	Solar light	180	99.17	This work
		UV light		86	
TiO_2_/activated carbon	50	Solar light	180	70	[79]
TiO_2_/cellulosic fiber	40	Solar light	150	83	[80]
Zr-WO_3_	20	UV light	210	73	[81]
Ag/ZnO	50	Solar light	240	83	[73]
		UV light		55	

## Data Availability

The raw data supporting the conclusions of this article will be made available by the authors on request.

## Supplementary Materials

The following supporting information can be downloaded at: https://www.mdpi.com/article/10.3390/nano15050358/s1, Figure S1. Geographic location of the experimental site in northern Algeria (latitude 36.39° N, longitude 2.42° E). Figure S2. Temporal variation of solar intensity throughout the experimental day. Figure S3. UV lamp spectra provided by the manufacturer. Figure S4. UV–vis reflectance spectra of pristine TiO_2_ and Au/TiO_2_ measured between 250 and 700 nm. Figure S5. Calibration curve of Paracetamol. Figure S6. TiO_2_ and Au-TiO_2_ photocatalytic activities of photodegradation of paracetamol under (a) sunlight irradiation and (b) UV lamp irradiation (C_PAR_ = 20 mg/L, C_catalyst_ = 1 g/L and free pH 6.8, for 180 min).
