# Supplementary material for: Sunlight-Induced Photocatalytic Removal of Paracetamol Using Au-TiO2 Nanoparticles
Source: Nanomaterials (Basel). 2025 Feb 26;15(5):358. doi: 10.3390/nano15050358 (PMC11901493; doi:10.3390/nano15050358)
Supplement: Supplementary file 1 [file nanomaterials-15-00358-s001.zip › nanomaterials-3442682-supplementary.pdf]

Supplementary Materials

Sunlight-induced Photocatalytic Removal of Paracetamol using Au-TiO_2 nanoparticles

L. Aoudjit [1,†], Joana M. Queirós [2,3,4,†,*] , A.S. Castro [4,5], Djamila Zioui [1], Noelia González-Ballesteros [6], S. Lanceros-Mendez [2,7,8,*] and P.M. Martins [3,4]

[1]Unité de Développement des Equipements Solaires, UDES /Centre de Développement des Energies Renouvelables, CDER, Bou Ismail, 42415, W. Tipaza, Algeria.
[2]Physics Centre of Minho and Porto Universities (CF-UM-UP) and LaPMET - Laboratory of Physics for Materials and Emergent Technologies, University of Minho, 4710-057, Braga, Portugal
[3]Centre of Molecular and Environmental Biology, University of Minho, 4710-057, Braga, Portugal
[4]IB-S – Institute for Research and Innovation on Bio-Sustainability, University of Minho
[5]Centre of Chemistry, University of Minho, 4710-057 Braga, Portugal
[6] Universidade de Vigo, Departamento de Química Inorgánica, 36310 Vigo, Spain
[7]BCMaterials, Basque Center for Materials, Applications and Nanostructures, UPV/EHU Science Park, 48940 Leioa, Spain
[8]Ikerbasque, Basque Foundation for Science, 48009 Bilbao, Spain
† These authors equally contributed to this work.
* Correspondence: authors

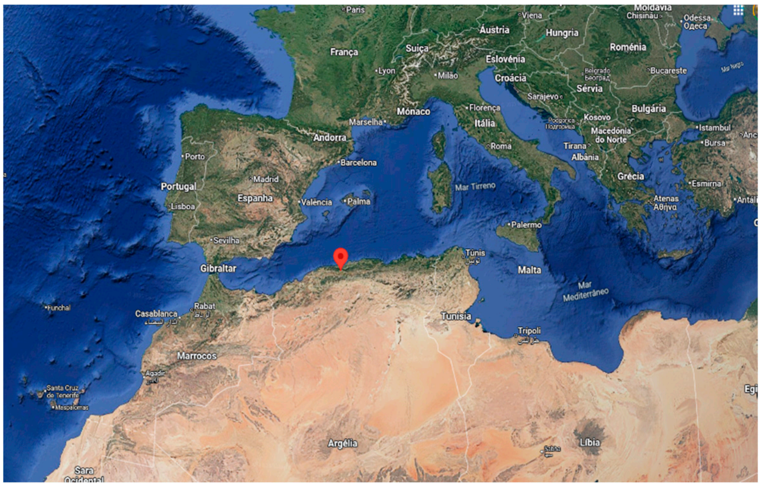

Figure S1. - Geographic location of the experimental site in northern Algeria (latitude 36.39° N, longitude 2.42° E).

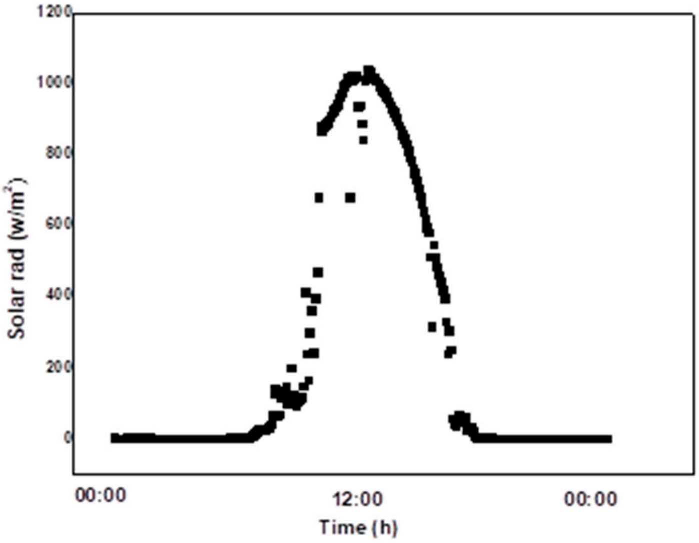

Figure S2. Temporal variation of solar intensity throughout the experimental day.

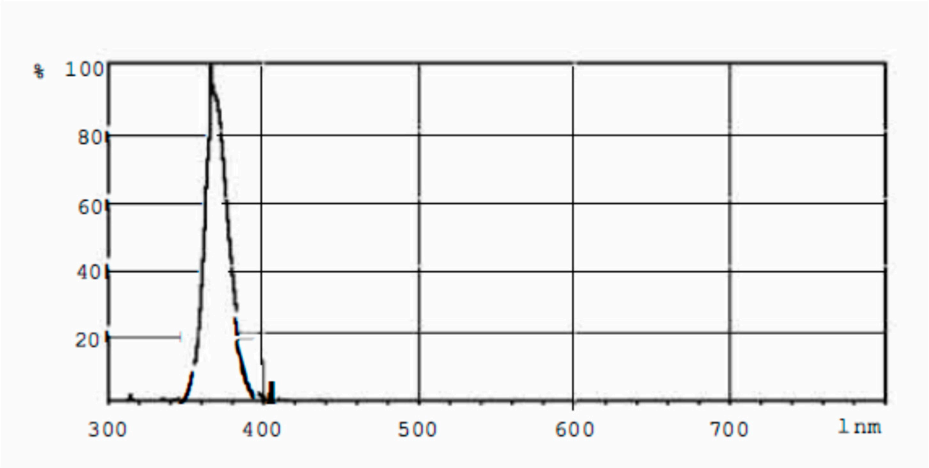

Figure S3. UV lamp spectra provided by the manufacturer.

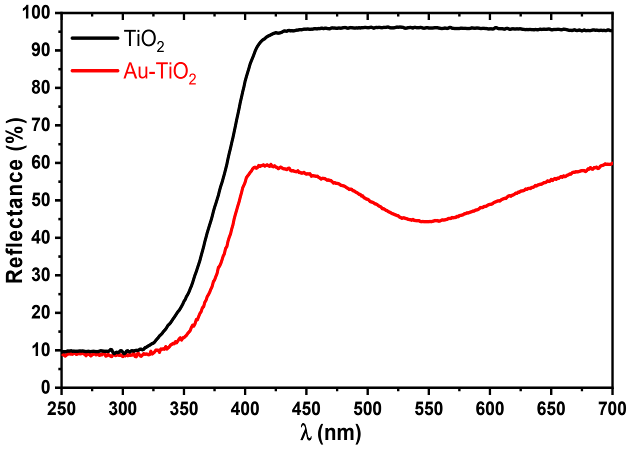

Figure S4. UV–vis reflectance spectra of pristine TiO_2 and Au/TiO_2 measured between 250 and 700 nm.

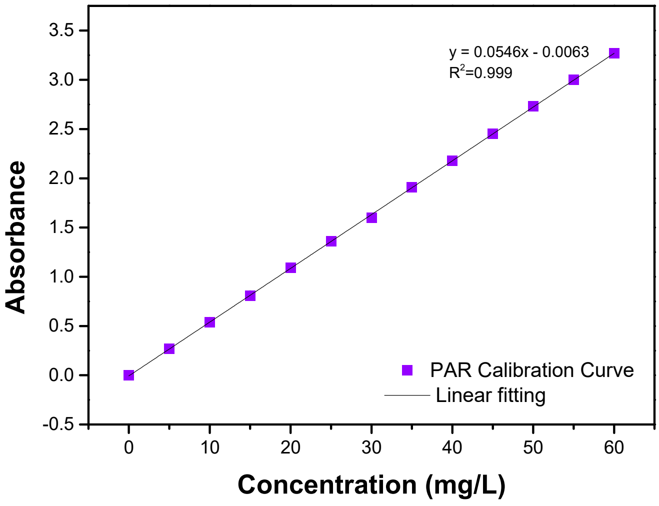

Figure S5. Calibration curve of Paracetamol.

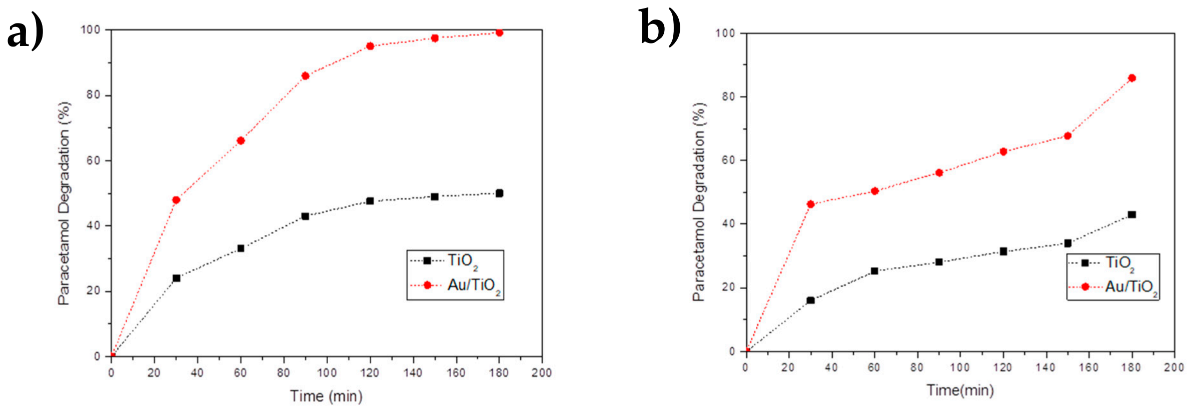

Figure S6. TiO_2 and Au-TiO_2 photocatalytic activities of photodegradation of paracetamol under **a)** sunlight irradiation and **b)** UV lamp irradiation (C_{PAR} = 20 mg/L, $C_{catalyst}$ = 1 g/L and free pH 6.8, for 180 minutes).
